# Recommendations for Interpreting and Reporting Silent Carrier and Disease-Modifying Variants in SMA Testing Workflows

**DOI:** 10.3390/genes13091657

**Published:** 2022-09-15

**Authors:** John N. Milligan, Laura Blasco-Pérez, Mar Costa-Roger, Marta Codina-Solà, Eduardo F. Tizzano

**Affiliations:** 1 Asuragen, A Bio-Techne Brand, Austin, TX 78721, USA; 2 Department of Clinical and Molecular Genetics, University Hospital Vall d’Hebron, 08035 Barcelona, Spain

**Keywords:** spinal muscular atrophy, carrier screening, diagnosis, *SMN1*, *SMN2*

## Abstract

Genetic testing for SMA diagnosis, newborn screening, and carrier screening has become a significant public health interest worldwide, driven largely by the development of novel and effective molecular therapies for the treatment of spinal muscular atrophy (SMA) and the corresponding updates to testing guidelines. Concurrently, understanding of the underlying genetics of SMA and their correlation with a broad range of phenotypes and risk factors has also advanced, particularly with respect to variants that modulate disease severity or impact residual carrier risks. While testing guidelines are beginning to emphasize the importance of these variants, there are no clear guidelines on how to utilize them in a real-world setting. Given the need for clarity in practice, this review summarizes several clinically relevant variants in the *SMN1* and *SMN2* genes, including how they inform outcomes for spinal muscular atrophy carrier risk and disease prognosis.

## 1. Spinal Muscular Atrophy Disease Etiology

Spinal muscular atrophy (SMA) is an autosomal recessive neuromuscular disease caused by loss of survival motor neuron 1 (*SMN1*) gene function and is a primary genetic cause of infant death [1]. SMA is a rare disease with a pan-ethnic incidence of ~1/11,000 live births and a high carrier rate of ~1/54 [2]. SMA is divided into clinical types based on the age of onset and maximum motor milestone achievement, with a gradient of phenotypes ranging from never sitting unassisted, with onset prior to six months of age, to adult-onset mild muscular weakness. Most SMA patients are classified into three main types in order of decreasing severity: type 1 (~60% of patients), type 2 (~30% of patients), and type 3 (~10% of patients). Rarer SMA types, such as type 0 and type 4, also exist [3,4,5].

**Figure 1:**
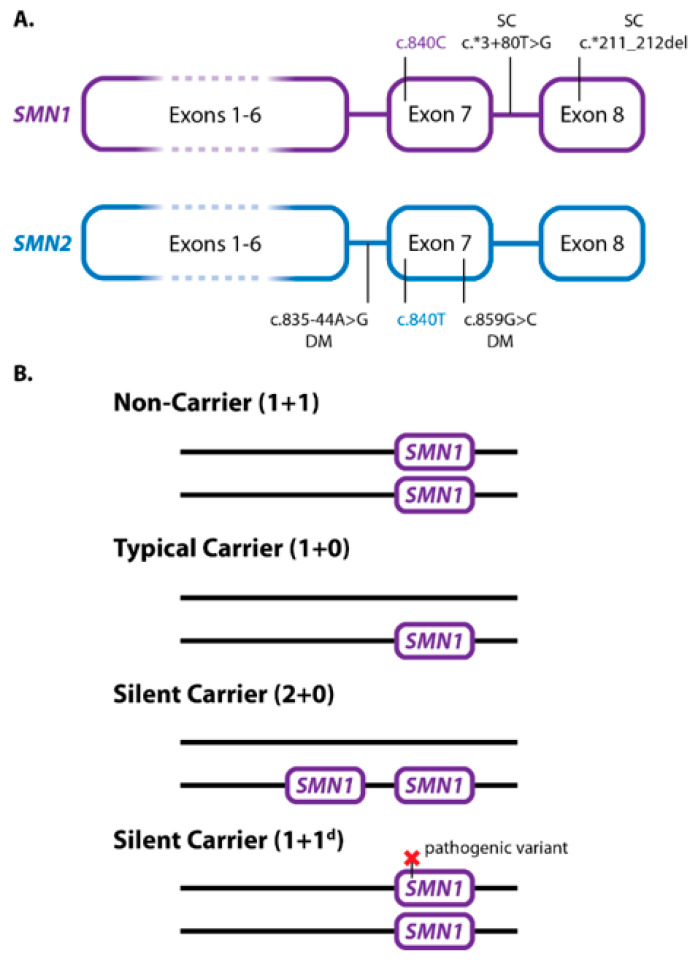
Genetics of *SMN1*, *SMN2*, and SMA Carriers. (**A**) Silent carriers and disease-modifying variants in *SMN1* and *SMN2*. Nucleotides at position c.840 in exon 7, typically used to distinguish *SMN1* and *SMN2*, are indicated by color (PSVs). Gene duplication variants in *SMN1* associated with 2 + 0 silent carriers are indicated by the letters SC. Common disease modifier variants in *SMN2* are indicated by the letters DM. (**B**) SMA carrier genetics. Non-carriers typically have one copy of *SMN1* on each chromosome. Typical carriers have only one *SMN1* copy, lacking *SMN1* on the other chromosome. Silent carriers (2 + 0) often have two copies of *SMN1* on a single chromosome, lacking *SMN1* on the other chromosome. Silent carriers can also have one copy of *SMN1* on both chromosomes but with a pathogenic variant in one copy.

Bi-allelic loss of the *SMN1* gene is the cause of disease in ~95% of patients with SMA. The remaining 5% of patients are compound heterozygotes, with an *SMN1* deletion on one chromosome and a loss-of-function point mutation in *SMN1* on the other chromosome. The vast majority (~98%) of SMA patients inherit the *SMN1* alterations from their parents [6,7]. SMA carriers lack a functional *SMN1* copy on a single chromosome and frequently have one functional copy on the other (1 + 0). However, a *cis* carrier genotype with two *SMN1* copies on a single chromosome (2 + 0), commonly referred to as a silent carrier, is also well-documented [8]. In one study examining a large North American population, the detection rate of SMA carriers using *SMN1* copy number alone varied from ~71% to 95% depending on ethnicity [9]. Most of the missed carriers were due to silent carriers (2 + 0) that cannot be resolved from wild-type (1 + 1) individuals solely based on copy number, since results would be 2 *SMN1* copies for both genotypes [9]. While gene conversion from *SMN2* to *SMN1* is known to occur and is one potential cause for the silent carrier (2 + 0) genotype [8], the clinical significance of gene conversions is not fully understood. Recent studies have shown that variants c.*3+80T>G and c.*211_*212del in *SMN1* (Figure 1A) are associated with *SMN1* duplication in many ethnic groups and their presence informs the risk of silent carrier *SMN1* genotypes (2 + 0) to varying degrees depending on ethnicity [10,11].

SMA disease severity inversely correlates with *SMN2* copy number, meaning the more copies of *SMN2*, the less severe the phenotype [5]. *SMN1* and *SMN2* differ in 16 paralogue sequence variants (PSVs) [12]. One PSV, c.840C>T, disrupts a splice enhancer that decreases the number of exon 7 containing mRNAs to 10–20%, which results in a significantly reduced amount of functional SMN protein compared to that made from a functional *SMN1* gene. However, due to complete homology with the *SMN1*-associated SMN protein sequence, *SMN2*-generated SMN protein levels offer a compensatory effect, thus resulting in lessened disease severity with increased *SMN2* copies. Though the *SMN2* copy number is vital for assessing disease severity, there are also a few variants known to be SMA disease modifiers. Specifically, c.859G>C in *SMN2* (Figure 1A) is linked to improved splicing efficiency of *SMN2* by 20%, which also leads to reduced disease severity [13,14]. Indeed, 44 SMA patients carrying the c.859G>C variant have been described, all of whom presented a milder phenotype than expected according to their *SMN2* copies. This variant has been described in various populations, showing a common haplotype that points towards a common ancestral origin [12]. Thus, *SMN1* is associated with molecular SMA diagnosis and carrier status, whereas *SMN2* is associated with the severity of the disease.

## 2. SMA Diagnostic and Carrier Screening Testing

Copy number analysis for *SMN1* and *SMN2* genes associated with SMA can be difficult, as the copy number of these varies much more than other regions within the genome. Furthermore, rapid turnaround time for SMA diagnostic testing is important for timely administration of therapies which halt neuron degeneration [15,16]. SMA genetic testing for *SMN1* and *SMN2* exon 7 copy numbers is accomplished using a variety of methods, including PCR followed by capillary electrophoresis (PCR/CE), quantitative PCR (qPCR), digital droplet PCR (ddPCR), multiplex ligation-dependent probe amplification (MLPA), and next-generation sequencing (NGS). These methods have recently been extensively described, including the strengths and weaknesses of each approach [17]. PCR-based systems are generally the fastest and simplest methods, though qPCR and ddPCR assays require separate reactions for each gene, and qPCR requires the generation of a standard curve, which can limit throughput. MLPA and PCR/CE both provide copy numbers for *SMN1* and *SMN2*, but MLPA has a longer and more complex workflow, requiring at least 24 h to complete as compared to PCR/CE, which can be completed in a few hours [17]. While PCR/CE is restricted to quantifying exon 7 and intron 7 from *SMN1* and *SMN2*, MLPA quantifies all exons in these genes, which can reveal partial gene deletions. NGS provides the most comprehensive analysis for variants, hybrid genes, and partial deletions, but the workflow can be laborious, time-intensive, and requires complex instrumentation. Furthermore, NGS analysis and interpretation requires significant hardware resources and bioinformatics expertise, especially for *SMN1* and *SMN2* analysis, given the high homology between the genes and high variability in potential copy numbers [18]. Recently, a more focused NGS method to analyze these genes provides full characterization of the *SMN* region in an affordable manner [19].

Traditionally, testing for *SMN1* exon 7 copy number alone is used for SMA diagnosis. However, a deletion of exon 8 alone has been reported in milder SMA types in two patients [20]. In addition, exon 8 information may have utility for the detection of hybrid genes, depending on the testing methodology [21,22]. Although typical *SMN1/2* hybrids involving exon 7 and exon 8 are the most common reported in the literature [6,23], hybrid genes may also be detected using other loci that differentiate *SMN1* and *SMN2*, for example, by comparing exon 7 and intron 7 [24] or involving intron 6 to exon 8 [19].

In addition to copy numbers, some methods are also able to detect variants in the *SMN1* and *SMN2* genes associated with silent carrier risk and disease severity, as detailed in the following sections. In short, the test methodology should balance the need for the right information to guide clinical care in the shortest possible timeframe with practical constraints such as the availability of instrumentation, personnel, and other resources.

## 3. SMA Carrier Genotypes, Testing, and Reporting

An SMA carrier is an asymptomatic individual lacking a functional copy of *SMN1* on one chromosome. Most SMA carriers have an *SMN1* deletion on one chromosome and one functional *SMN1* copy on the other (1 + 0), representing a heterozygous deletion (Figure 1B). Silent carriers, in contrast, have a (2 + 0) genotype, whereas others may have another type of pathogenic variant in *SMN1* on one chromosome and two *SMN1* copies (1^d^ + 1), or rarer genotypes with higher *SMN1* copy numbers (1^d^ + 2, 3 + 0) [8]. Due to these multiple genotypes, the detection rate of SMA carriers using the *SMN1* copy number alone to detect (1 + 0) genotypes varies from ~71% up to 95% depending on ethnicity [9]. Thus, there is a proportion of false-negative results for carrier status when reporting only the *SMN1* copy number. Residual carrier risk estimations based on the *SMN1* copy number alone have been calculated by compiling results across multiple studies and ethnicities (Table 1, first four columns) [25]. Since the total *SMN1* copy number is used to assess carrier risk, the limitations of such testing, specifically the inability to detect silent carriers using *SMN1* copy number alone, should be described when reporting results [8].

In addition to the *SMN1* copy number, data has shown that the presence of *SMN1* gene duplication variants c.*3+80T>G in intron 7 and c.*211_*212del in exon 8 (Figure 1A) can be indicative of the silent carrier (2 + 0) genotype in many ethnicities [10,11]. Several test methods can detect these variants, including MLPA (P-460), NGS, PCR/Sanger, and PCR/CE [12,18,19,24,26,27]. Typically, these variants co-occur [10]; however, individuals with only one of the two variants have been identified [11]. Detection of either c.*3+80T>G or c.*211_*212del alone is generally considered indicative of *SMN1* gene duplication, and thus associated with increased silent carrier risk [10]. However, c.*211_*212del in exon 8 has been detected in *SMN2* hybrid genes in SMA patients with no copies of *SMN1*, indicating that it is possible that an isolated occurrence of either can be associated with a hybrid gene [11].

In response to characterization of the *SMN1* gene duplication variants across multiple ethnicities, guidelines have been updated to reflect that these variants improve residual risk estimates [28]. Table 1 (last two columns) summarizes these results across several studies, which can be used to provide an estimate of residual risk based on ethnicity. The impact of these variants has not been evaluated in all ethnicities, and some studies show varying residual risk levels within an ethnicity [10,18,29]. This is likely due to both the broad range of ethnic backgrounds included in each category and the fact that ethnicities are often self-reported, which creates ambiguity in how these groups are classified and reported [30]. Consequently, the numbers shown here represent risk estimations from studies with the largest number of individuals analyzed for each ethnicity, recognizing that while these are the best estimations available, they are not exact figures. Continued research is needed to further refine both diagnostic interpretations and residual risk values for different genetic ancestries, so literature should be reviewed regularly [31].

The absence of these gene duplication variants does not rule out the possibility of a carrier (2 + 0) genotype, nor does their presence definitively diagnose silent carriers across different ancestries. In these cases, the analysis of copy number in the progenitors of the carrier under study would help to determine the cis or trans configuration of *SMN1* genes, though this implies extra testing that is not always possible [11]. Nevertheless, resolution of *SMN1* gene duplication variants modifies the residual risk of SMA carrier status in all ethnicities studied to date (Table 1). Therefore, co-occurrence of these variants with two copies of *SMN1* indicates increased carrier risk, while absence of the variants with two copies of *SMN1* indicates reduced carrier risk compared to using *SMN1* copy number alone, regardless of ethnicity [10,11,18,28,29].

For reporting purposes, *SMN1* gene duplication variant information is relevant only when two copies of *SMN1* are present; variant interpretation is not necessary when a one *SMN1* copy carrier genotype (1 + 0) is identified through *SMN1* copy number testing. Furthermore, when three or more copies of *SMN1* are present, interpretation of these variants is unnecessary given the extremely low likelihood of being a carrier [25]. In cases where ethnicity is unknown, uncertain, or unreported, a range of possible risk values may be provided and discussed in counseling patients, while noting that risk varies depending on ethnicity and, more specifically, ancestry [30]. To clarify potential reporting, examples of *SMN1* copy number and gene duplication variant status results in a carrier screening setting are provided in Table 2 based on available guidelines [8,28]. See also Prior et al. 2011 for an example report [8].

Since gene conversions are another mechanism that can lead to silent carriers [8], evidence of conversion from *SMN2* to *SMN1* (*SMN1/2* hybrids) could inform silent carrier risk. However, this possibility has not been sufficiently investigated clinically, and hybrid genes have a variable gene architecture [32]. As a result, there is insufficient evidence to determine carrier risk based on hybrid genes.

## 4. Disease Prognosis Genotypes, Testing, and Reporting

While the *SMN2* copy number is not relevant for the diagnosis of SMA, guidelines recommend that *SMN2* copy number results be reported to inform prognosis and treatment decisions [17,33,34,35]. The *SMN2* copy number is strongly correlated with SMA type, but the copy number alone is not sufficient to predict SMA type. This limitation should be clearly communicated when reporting *SMN2* copy number results.

Additionally, the c.859G>C variant is a positive disease modifier associated with reduced disease severity and improved prognosis. Several test methods can detect this variant, including NGS, specific PCR/Sanger, and PCR/CE [19,24]. Evidence indicates that c.859G>C improves *SMN2* splicing, exon 7 inclusion, and full-length SMN protein production, leading to improved phenotypic outcomes [13,14]. For instance, while 90% of individuals with SMA and two copies of *SMN2* exon 7 typically have SMA type 1, individuals with SMA that have two copies of *SMN2* exon 7 and the c.859G>C variant typically have SMA type 2 or type 3, with no known cases of SMA type 1 in individuals where this variant is present [13,14,33]. A similar effect has been observed in patients with three copies of *SMN2* exon 7 and the c.859G>C variant, typically resulting in SMA type 3 [12,33]. The number of *SMN2* copies with c.859G>C also correlates with phenotype, with multiple copies leading to milder phenotypes [12]. While the c.859G>C variant has not been reported in patients with one or four copies of *SMN2*, available evidence suggests that any individual with this variant would have a milder phenotype than expected based on *SMN2* copy number alone.

In addition to c.859G>C, another positive modifier known as c.835-44A>G has been described (Figure 1A), albeit with limited investigation in SMA patients to date. This variant is one of the PSV differentiating *SMN1* from *SMN2*, and its presence in intron 6 of *SMN2* increases the inclusion of exon 7 [36]. This modifier can be detected with specific PCR/Sanger or NGS methods [12,19]. Other putative positive and negative disease modifiers have been described [15,17,32]. However, these variants have been identified only in a small number of patients without a clear genotype-phenotype correlation [19].

Aside from SNP and INDEL variants that impact disease prognosis, several recent publications have mentioned *SMN1/2* hybrids as another positive disease modifier [15,37,38,39]. These hybrid genes arise when *SMN1* is partially converted to *SMN2* or vice versa. Since they retain elements of *SMN1*, some hybrids can increase exon 7 inclusion in SMN mRNAs compared to typical *SMN2*, producing greater quantities of full length SMN protein that lead to a milder phenotype [37,38]. However, *SMN1/2* hybrids are heterogeneous, and their impacts on full-length SMN transcript and protein quantity are likely dependent on which *SMN1* elements are retained [37]. More data are needed to inform the interpretation of hybrid genotypes beyond the general observation that *SMN1/2* hybrids can be associated with milder phenotypes.

For reporting purposes, likely prognosis can be interpreted using *SMN2* copy number alone when disease-modifying variants are not detected, noting that the correlation between genotype and phenotype is not absolute [8,34,35]. A positive result for c.859G>C may be reported as a marker associated with reduced severity and/or improved prognosis in comparison with the typical presentation based on the *SMN2* copy number genotype. To clarify probable SMA types based on *SMN2* copy number and c.859G>C, a summary of published treatment guidelines and peer-reviewed studies is provided in Table 3. This prognostic information is relevant only for individuals diagnosed with SMA. Examples for reporting *SMN2* copy number and c.859G>C status when providing test results are provided. Other disease modifier variants such as c.835-44A>G or the presence of *SMN2* hybrids can be reported when further research genetic studies are performed, mainly in discordant patients [15,17].

## 5. Newborn Screening for SMA

With multiple treatment options available and compelling data showing the value of early treatment to maximize patient benefit, SMA newborn screening (NBS) has become an increasing priority. In the US, this screening is included in the RUSP (Recommended Uniform Screening Panel) and other NBS recommendations [34]. In the same line, the SMA NBS Alliance promotes the implementation of NBS in all of Europe by 2025 (www.sma-screening-alliance.org/ (accessed on 12 September 2022)).

In SMA NBS, *SMN1* is the primary indicator of disease status. Given the throughput and cost restrictions necessary for NBS, testing is often limited to the presence or absence of *SMN1* exon 7 using DNA isolated from dried blood spots (DBS) and is frequently combined with testing for severe combined immunodeficiency (SCID) in a single assay [34,40]. When positive screening results are identified, follow-up testing is performed to confirm diagnosis and obtain *SMN2* copy number results to infer disease prognosis. However, recent studies have provided data supporting the reporting of *SMN2* copy numbers along with initial screening results, as it is beneficial for SMA patients with two copies of *SMN2* where treatment timing is most crucial [16]. Others have suggested that disease modifier variant testing is also important to further refine the likely prognosis for SMA patients identified through NBS with two or three copies of *SMN2* [17]. As NBS programs and our understanding of the intersection of screening and treatment continue to expand, it is likely that NBS testing will move toward providing as much genetic information as possible to maximize treatment benefits in newborns with SMA [41]. As the complexity of NBS is increasing, genetic programs in newborns should come along with adequate pre-test genetic counseling to provide more precise information to the families.

## 6. Conclusions

While understanding of the impact of *SMN1* and *SMN2* variants on SMA carrier status and disease prognosis continues to evolve, a solid foundation of clinical studies demonstrates the utility of identifying several variants in addition to copy numbers. More specifically, when variants predicting *SMN1* copies in cis are present, it is possible to adjust the risk of silent carrier status, which can help inform reproductive decisions for couples. Additionally, disease modifier testing can improve prognostic predictions in individuals diagnosed with SMA, explaining some of the discrepancies between observed *SMN2* copy numbers and expected SMA disease progression. The information provided by these variants can benefit laboratories and clinicians interested in providing more accurate SMA carrier screening and prognostic predictions.

## Figures and Tables

**Table 1:** Residual SMA carrier risk estimates by ethnicity based on *SMN1* copy number and gene duplication variant status. Carrier frequency represents carrier risk without testing by ethnicity. Subsequent columns estimate residual risk based on *SMN1* copy number alone. The last two columns estimate the residual risk with two copies of *SMN1* with additional information on the presence of *SMN1* gene duplication variants (*SMN1* c.*3+80T>G and c.*211_*212del), where “positive” indicates presence of one or both variants, and “negative” indicates absence of both variants. Values are rounded to the nearest integer. Asian includes groups with South Asian and East Asian ancestry.

Ethnicity	Carrier Frequency	2 Copies *SMN1* Exon 7	3 Copies *SMN1* Exon 7	2 Copies *SMN1*, Variant Status “Negative”	2 Copies *SMN1*, Variant Status “Positive”
Ashkenazi Jewish	1:56 ^a^	1:514 ^a^	1:5899 ^a^	1:580 ^b^	~1 ^b^
Asian	1:50 ^a^	1:719 ^a^	1:5185 ^a^	1:779 ^c^	1:57 ^c^
African American/Black	1:71 ^a^	1:132 ^a^	1:6997 ^a^	1:375 ^d^	1:39 ^d^
Caucasian/European	1:45 ^a^	1:604 ^a^	1:4719 ^a^	1:814 ^c^	1:12 ^c^
Hispanic	1:83 ^a^	1:641 ^a^	1:7574 ^a^	1:906 ^d^	1:99 ^d^
Spanish	1:40 ^e^	1:781 ^e^	Not Reported	1:888 ^e^	~1 ^e^
Israeli Jewish	1:38 ^a^	1:450 ^a^	1:4004 ^a^	Not Reported	Not Reported
Asian Indian	1:50 ^a^	1:428 ^a^	1:5252 ^a^	Not Reported	Not Reported
Iranian	1:16 ^a^	1:96 ^a^	1:1604 ^a^	Not Reported	Not Reported

Data for risk estimates adapted from references as indicated with letters. a: [25]. b: [10]. c: [18]. d: [29]. e: [11].

**Table 2:** Carrier Results Interpretation Examples. The examples provided here are interpretations based on relevant guidelines [8,11] and literature [10,11,18,25,29]. When interpreting and presenting results, all relevant local guidelines and regulations should be followed.

Example Results	*SMN1* Copies	c.*3+80T>G	c.*211_ *212del	Interpretation
Case 1	1	Not indicated	Not indicated	Carrier The *SMN1* copy number indicates a carrier of SMA. Genetic counseling is recommended and carrier testing should be made available to other at-risk family members.
Case 2	2	Positive	Negative	Increased Carrier Risk The *SMN1* copy number is two, ruling out a typical carrier genotype (1 + 0). However, the presence of one or more variants indicates an increased risk of being a silent carrier. The residual risk of SMA carrier status based on genotype alone is between 1:99 to ~1 depending on ethnicity. Ethnic-specific risk values based on these results are provided (see Table 1, last column). Parental testing should be considered to elucidate the presence of a silent carrier (2 + 0). Genetic counseling is recommended and carrier testing should be made available to other at-risk family members.
Case 3	2	Positive	Positive	Increased Carrier Risk Refer to Case 2 for example language.
Case 4	2	Negative	Negative	Reduced Carrier Risk The *SMN1* copy number and variant status indicate reduced, but not eliminated, carrier risk. The residual risk of SMA carrier status based on genotype alone is between 1:375 and 1:906 depending on ethnicity. Ethnic-specific risk values based on these results are provided (see Table 1, 2nd to last column). Genetic counseling is recommended.
Case 5	3	At genetic counselor’s discretion	At genetic counselor’s discretion	Reduced Carrier Risk The *SMN1* copy number indicates a significantly reduced, but not eliminated, carrier risk. The residual risk of SMA carrier status based on genotype is low. Ethnic-specific risk values based on these results are provided (see Table 1, Column 4). Genetic counseling is recommended.

**Table 3:** Likely SMA prognosis based on *SMN2* copy number and variant status. *SMN1* copy numbers are presumed to be 0, consistent with diagnosis. Genotypes not referenced below (e.g., 3 copies *SMN2* with two or more c.859G>C alleles) have not yet been reported. The reporting examples provided here are interpretations based on consensus recommendations published by the American College of Medical Genetics (ACMG), Cure SMA, and the SMA Care group [8,34,35], as well as other relevant guidelines and literature [13,14,17,33]. For recommendations on follow-up testing and management of SMA cases as well as probability estimations of SMA type based on results, see [17]. When interpreting and presenting results, all relevant local guidelines and regulations should be followed.

SMN2 Copy Number	c.859G>C Variant Status	Interpretation and Reporting Example
1	Negative	SMA (Type 0 probable) ^a^ Most individuals with SMA and one *SMN2* copy present with Type 0 congenital disease. While the relationship between *SMN2* copy number and disease outcomes is strongly correlated, it is not absolute, and individual exceptions do occur. Genetic counseling is recommended.
2	Negative	SMA (Type 1 probable) ^a^ Most individuals with SMA and two *SMN2* copies present with Type 1 SMA. Refer to other examples with Negative c.859G>C Variant Status for example language.
2	Detected in one copy	SMA (Type 2/3 probable) ^b,c^ Whereas most individuals with SMA and two *SMN2* copies present with Type 1 SMA, the presence of the c.859G>C variant in one *SMN2* copy is associated with reduced severity consistent with SMA Type 2/3. Genetic counseling is recommended.
2	Detected in two copies	SMA (Type 3/4 probable) ^c,d^ Whereas most individuals with SMA and two *SMN2* copies present with Type 1 SMA, the presence of the c.859G>C variant in two *SMN2* copies is associated with reduced severity consistent with SMA Type 3/4. Genetic counseling is recommended.
3	Negative	SMA (Type 2/3 probable) ^a^ Refer to other examples with negative c.859G>C variant status for an example language.
3	Detected in one copy	SMA (Type 3 probable) ^c,e^ Whereas most individuals with SMA and three *SMN2* copies present with Type 2/3 SMA, the presence of the c.859G>C variant in one *SMN2* copy is associated with reduced severity consistent with SMA Type 3. Genetic counseling is recommended.
≥4	Negative	SMA (Type 3/4 probable) ^a^ Refer to other examples with negative c.859G>C variant status for example language.

Interpretation of phenotype and source data adapted from references as indicated with letters. a: [17,34]. b: [13,14,33]. c: [12] d: [42] e: [33].

## Data Availability

Not applicable.
